# Gaming on an Immersive Virtual Reality Platform to Ameliorate the Level of Anxiety in Patients Undergoing Congenital Heart Disease

**DOI:** 10.7759/cureus.50694

**Published:** 2023-12-17

**Authors:** Mansee Dangare, Vaishnavi Yadav

**Affiliations:** 1 Cardiovascular and Respiratory Physiotherapy, Ravi Nair Physiotherapy College, Datta Meghe Institute of Higher Education and Research, Wardha, IND

**Keywords:** double outlet right ventricle, chest physiotherapy, anxiety, behavioral problems, virtual reality

## Abstract

The double outlet right ventricle (DORV) is an unusual cardiovascular abnormality that is present at birth. The pulmonary artery and the aorta, the cardiovascular system's two major arteries, link to the right ventricle in the DORV. The coronary artery links to the right ventricular, while in an ideal heart, the aorta connects to the left ventricle. DORV is an issue as the right ventricle delivers blood that is low in oxygen, subsequently distributed all around the human being. DORV is always connected with a further cardiac ailment known as a ventricular septal defect (VSD). Behavioral issues are quite prevalent in children. Emotional-obsessive-compulsive disorder, sadness, stress, social fear, and developmental problems are among them. Virtual reality (VR) is a stimulating experience that uses position tracking and three-dimensional (3D) near-eye presentation to give the participant a realistic view of a virtual environment. VR applications involve enjoyment (especially video games). A nine-year-old female complained of a tingling sensation over her right palm. She also had right-side weakness and convulsions, which were restricted to the dominant side. After investigation and evaluation of the condition, it is a known case of congenital heart defects and was planned for surgical correction. The study aims to provide information on an instance of DORV, transposition of great arteries, VSD, and pulmonary stenosis with resolved brain abscess by giving VR video games to reduce anxiety in patients undergoing an operation.

## Introduction

Congenital heart disease (CHD) refers to structural anomalies of the cardiovascular system that develop prior to childbirth. Following pregnancy, these abnormalities manifest in the growing fetus inside the uterus. These include anomalies of the cardiovascular muscle, a narrowing of defects of the atrial and ventricular divides flaws of the heart valves, and an opening in the heart wall that results in a fault in blood flow, cardiac arrest, and eventually death. Double outlet right ventricle (DORV) is a type of CHD [1].

Virtual reality (VR) is an effective device that people may use to learn new things that will improve their mental wellness. Through the use of interactive computer-produced worlds, immersive VR replaces genuine sensory experiences with digitally generated ones, giving users the impression that they are truly in brand-new, life-sized surroundings [2]. An immersive VR device relies heavily on perception via natural movement. If high degrees of participation are attained for circumstances that worry individuals, VR offers incredible opportunities to assist individuals in overcoming psychological difficulties [3]. Strategies that empower people to implement these kinds of adjustments in everyday life are the most effective [4].

VR gaming has the ability to significantly reduce stress and enhance the quality of life (QOL) for those with CHD. Because VR settings are immersive and participatory, people may escape the difficulties of their health issues and partake in a fun and healing activity [5]. VR gaming offers a novel means of enjoyment and diversion for those with CHD, who frequently encounter physical constraints and difficulties. Numerous VR games include soft motions or exercises that motivate players to perform mild exercise, which improves cardiac fitness and blood flow. Reduced stress and physical exercise have a twofold effect that supports cardiovascular fitness and improves patients' psychological resiliency in addition to enhancing their medical therapy [6].

With its ability to transport users to different dimensions through immersive and captivating situations, VR has become an effective method in the relief of stress for users. It can temporarily distract people from real-life problems by granting them a sensation of presence in a computer-generated world [7]. Because VR is immersive, it stimulates a variety of senses and produces an engrossing experience that can induce calm and happy feelings. VR offers a distinctive and customized method of stress relief, whether through tranquil virtual scenes from nature, meditations, or fun games [8]. We report a case of DORV with ventricular septal defect (VSD) repaired with bilateral bidirectional Glenn shunt (BDG) surgery and the crucial role of VR in alleviating the level of anxiety in patients who have CHD.

## Case presentation

A nine-year-old female patient was brought by her parents with the complaint of cough with sputum and cold. The patient also complained of a tingling sensation over the right palm for one day, which was associated with exertion and in a resting position for a long time, right side eye pain for seven days, associated with exertion headache more often in the morning after waking up from sleep and macular lesions over the body. She was a known case of DORV with transposition of great arteries and VSD with pulmonary stenosis. Previously, the patient had surgery for a tubercular brain abscess in the left temporoparietal lobe in April 2022. Then, after nine months, the patient had right-side weakness and convulsions, which were restricted to the right arm. The patient was brought to the Acharya Vinoba Bhava Rural Hospital, where an investigation, like chest radiography and 2D Echocardiography (2D Echo) was done. The patient had been advised surgery for a DORV with transposition of great arteries, VSD with pulmonary stenosis. She was admitted to the cardiac and thoracic operation unit on March 24, 2023, where she was observed and treated for bilateral BDG on March 31, 2023. She was moved into the cardiovascular and thoracic surgery incentive care unit and assigned to physiotherapy on the first surgical day.

Clinical findings

The examination was done by taking consent from the patient. The patient was alert, cooperative, and oriented to time, place, and person. On examination, there is a bandage on the front side of the thorax. She rated her pain at the site of the suture as 4/10 at relaxation and 7/10 while doing movements such as upper limb motion and coughing on the Numerical Pain Rating Scale (NPRS). On observation, the patient's build was mesomorphic. Vitals include the patient being afebrile, having an HR of 78 beats per minute, being intermittently unstable, RR being 22 breaths per minute, and having a BP of 110/80 mmHg. On systemic examination, heart sounds S1 and S2 were heard, and apical impulses at the fifth intercostal space. Auscultation revealed air entry bilaterally equal. The complete incident's timeframe is depicted in Table 1.

**Table 1:** Patient timeline BDG: Bidirectional Glenn shunt; CHD: Congenital heart disease

Operated for tubercular brain abscess	April 2022
First-time occurrence of symptoms	November 26, 2022
Admitted to a government hospital	November 29, 2022
Diagnosis of CHD	December 1, 2022
Right-hand weakness	January 1, 2023
Recurrence of symptoms	March 21, 2023
Date of admission	March 24, 2023
Date of BDG shunt surgery	March 31, 2023
Date of physiotherapy assessment and management	April 1, 2023
Date of discharge	April 11, 2023

Diagnostic assessment

**Figure 1:**
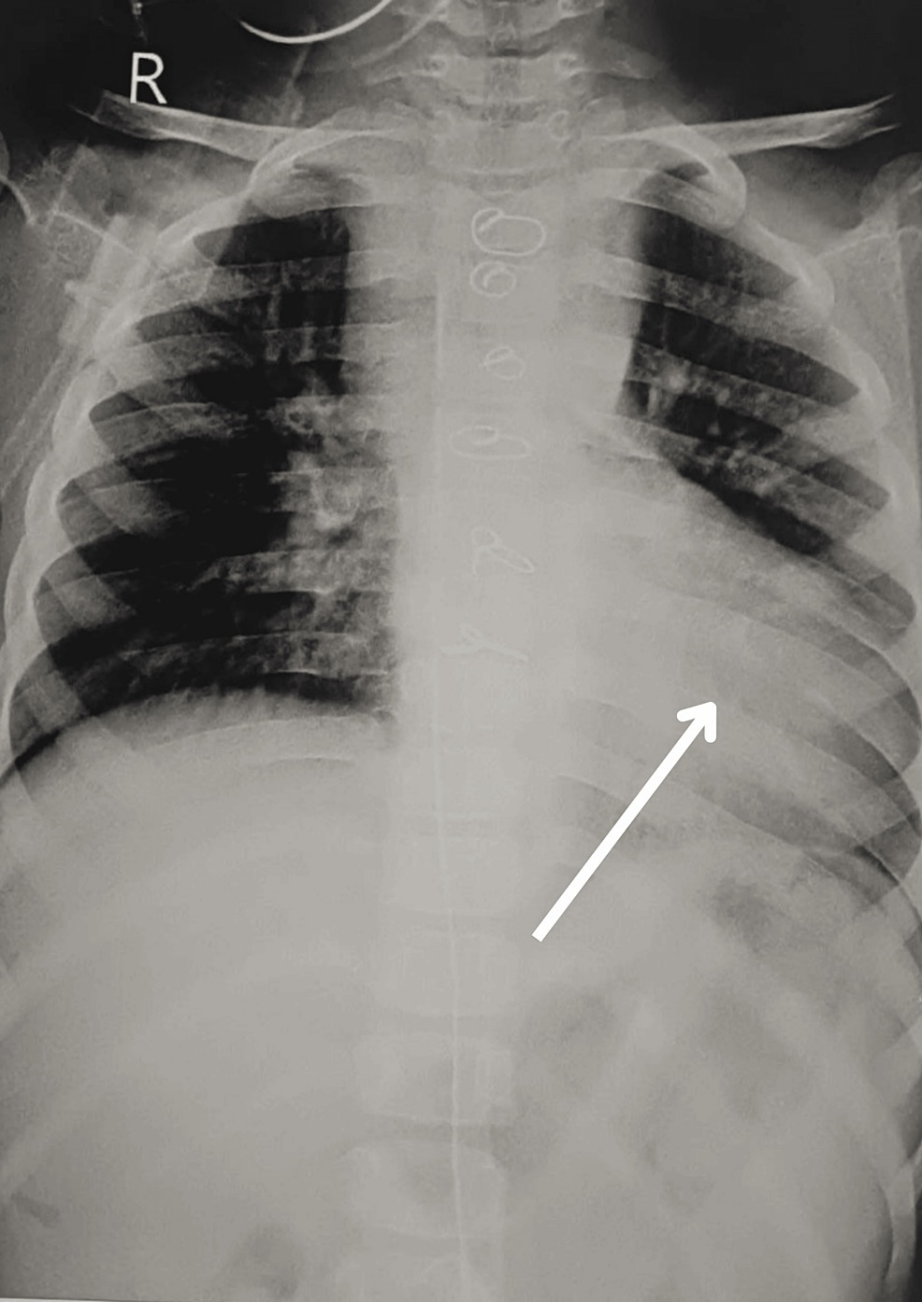
Chest X-ray The white arrow indicates cardiomegaly

Chest X-ray shows the bronchovascular markings over the right middle and lower zones. Hilar markings were present. Cardiomegaly is seen, and obliteration of cardio-phrenic and costo-phrenic angle in a postoperative chest X-ray is shown in Figure 1. Magnetic resonance imaging brain reveals gliotic changes in the left temporal-parietal region with ex-vacuo dilation of the ipsilateral lateral ventricle, causing a midline shift of 3 mm towards the left side with prominence of left sulco-gyral spaces and Sylvian fissure. 2D Echo report revealed DORV with transposition of the great arteries, VSD, pulmonary stenosis and ejection fraction was 60%. Electrocardiography (ECG) showed a typical sinus pattern, right ventricular hypertrophy, and possible lateral interaction. The confirmed diagnosis was DORV with transposition of great arteries with a VSD, and pulmonary stenosis was confirmed based on various diagnostic assessments and investigations.

Medical management

The patient was managed medically with antibiotics, nonsteroidal anti-inflammatory drugs, and anticoagulants, as depicted in Table 2.

**Table 2:** Medication dosage and duration Tab: Tablet; Syp: Syrup; mg: Milligrams; ml: Milliliters; BD: Twice a day; OD: Once a day; TDS: Thrice a day

Medication	Dosage	Duration
Tab. Gabapentin	100 mgs	TDS
Syp. Vacparcin	2 ml	BD
Tab. Paracetamol	250 mgs	TDS
Tab. Furosemide	10 mgs	BD
Tab. Aspirin	50 mgs	OD
Tab. Lansoprazole	150 mgs	BD

Therapeutic intervention

**Figure 2:**
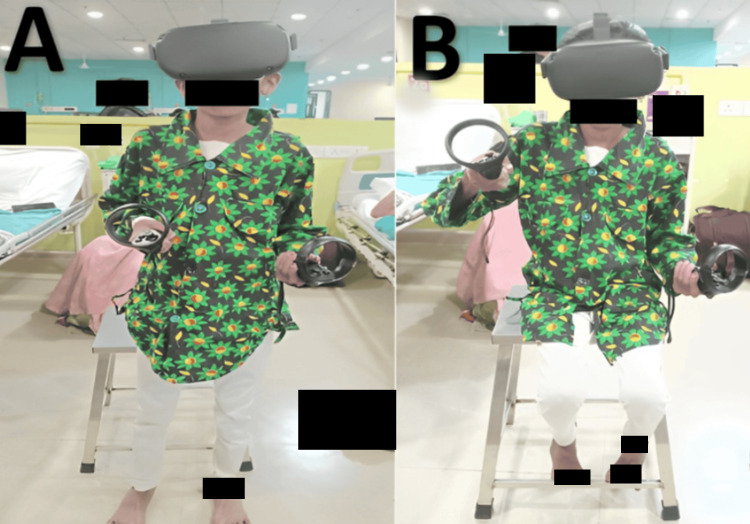
A patient using VR (A): In standing position and (B): In sitting position VR: Virtual reality

The goal-oriented chest physiotherapy protocol is depicted in Table 3. The therapy was performed under the observation of a physiotherapist. VR Oculus boxing game is shown in Figures 2A, 2B.

**Table 3:** Goal-oriented physiotherapy protocol cc: Cubic centimeter; ROM: Range of motion; Reps: Repetition; VR: Virtual reality

Sr. No	Physiotherapy goals	Therapeutic intervention	Treatment regimen
1.	To inform the patient and his relatives regarding the present medical condition	Patients and relatives must be aware of the need for a well-planned treatment and fitness routine, as well as the illness itself.	Teaching for earlier mobility, placement of limbs, and resumption of tasks associated with everyday routine
2.	Encourage calm and minimize pain after surgery	Place the patient in a semi-fowler's posture with the head of the bed raised to thirty degrees and the knees and hips slightly bent. The chest cut is less strained as a result of the location.	For 10-15 minutes daily
3.	To encourage airway clearance	Manual assisted huffing and coughing technique with splinting	4-8 days
4.	Maintain adequate circulation in the lower extremities to prevent deep vein thrombosis and pulmonary embolism	Active exercises of the lower extremities with emphasis on ankle pumping were started on the first day of surgery.	A single set of ten repetitions two times each day
5.	To improve lung expansion	Thoracic expansion with upper limb coordination exercises and incentive spirometry responses were given visibly using various color balls having red, yellow, and blue indicating 600 cc, 900 cc, and 1200 cc, respectively.	A single set of ten repetitions two times each day and 10 reps x 2 sets every 2 hours, respectively.
6.	Enhance your respiration rhythm as well as heart rate	Long inhalation exercise	A single set of ten attempts. Three to four times per day
7.	Recover shoulder full range of movement	Relaxation activities for the shoulder region began on the first day after the operation (including shoulder shrugging or shoulder circles), followed by active shoulder exercises on subsequent days after surgery, depending on the patient's tolerance, until complete active ROM was reached.	A single set of 10 repetitions two times each day
8.	Avoid postural problems	While the patient is in bed on the initial postoperative day, emphasize symmetric trunk posture and placement maintenance of a range of motion with passive movement.	Positioning two hourly. ROM exercise - 10 reps x 1 set
9.	To improve functional recovery and exercise tolerance	Ambulation and stairclimbing	Walk within a hall (one round)
10.	To reduce child anxiety and depression	Using the VR Oculus boxing game	For 10-15 min

Outcome measures

**Figure 3:**
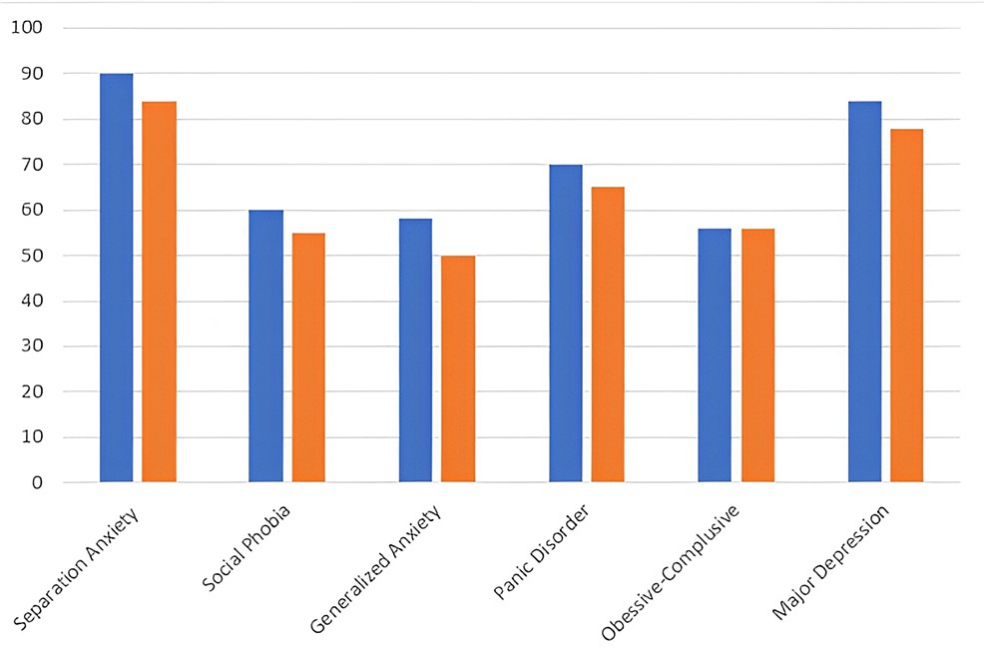
RCADS graph (Blue: Pre; Orange: Post) RCADS: Revised Child Anxiety and Depression Scale; Pre: Preoperative; Post: Postoperative

The outcome measure of the Revised Child Anxiety and Depression Scale (RCADS) is given in Table 4, RCADS graph is shown in Figure 3, and NPRS is depicted in Table 5. RCADS is a self-reported questionnaire that is used to measure depression and anxiety in children and adolescents.

**Table 4:** The Revised Child Anxiety and Depression Scale (RCADS) POD: Postoperative day

The Revised Child Anxiety and Depression Scale	POD 4	POD 15
Separation anxiety	90	84
Social phobia	60	55
Generalized anxiety	50	50
Panic disorder	70	65
Obsessive-compulsive	62	56
Major depression	82	78

**Table 5:** NPRS POD: Postoperative day; NPRS: Numerical Pain Rating Scale

NPRS	POD 4	POD 15
On rest	6/10	2/10
On activity	8/10	4/10

## Discussion

Participatory VR can potentially improve cognitive behavioral therapy among kids experiencing antisocial behavioral issues. It enables youngsters to practice interactions with others in a personally customized manner without the hazards associated with collective therapy [8,9]. This single-case experimental design experiment looked at the effectiveness of a VR biofeedback game (DEEP), as a tool for reducing stress and disrupted teaching activities in teenagers in a particular school context. The primary goal of the research was to see how playing DEEP affected everyday experiences with anxiousness and problematic classroom conduct. The findings showed a slight decrease in anxiety levels following the start of DEEP on an individual basis [10,11]. It was discovered that throughout the practice of therapy with VR, participants could walk with greater mobility, spontaneity, safety, and enjoyment, even in the presence of drains [12]. People going through heart surgery may exhibit changes in respiration dynamics due to trauma to the center of the chest and pain, leading to decreased lung volumes, in addition to ventilation/perfusion dysfunction, and a change in breathing habits throughout the post-surgery period [13].

According to the study, the application of VR may be a strategy for improving the recovery procedure since it is a platform that promotes more significant involvement by patients, encouraging happiness and enjoyment in its practice and encouraging a state of thinking and role to its recovery by allowing it to achieve its full potential faster and more playfully inside the medical facility. The application of VR increases these patients' drive and dedication, resulting in improved motor development, physical capability, and overall QOL. According to the findings, VR therapy successfully reduced lung conditions postoperatively and restored autonomy in coronary artery bypass patients [14,15].

The VR program employed in this instance relieved discomfort by diverting the patient's focus away from the source of the agony. The psychological impact of involvement in the virtual world provided by VR technology is supposed to be relieving pain. Another way that the VR program alleviated pain in this scenario would be to create a calming environment. The patient's emotion influences pain perception. The impacts of VR on diversion and calm could reduce feelings of pain in patients synergistically, improving healing. The VR programs utilized in this study were created to simulate a beach and sea environment. As a result, by offering patients excursion experiences and preserving their drive to undertake repetitious training at home, which can be tough to sustain, VR might be able to induce a positive mental shift in patients and, as a result, enhance their overall QOL [16,17].

The purpose of the study was to see how a VR distraction strategy affected discomfort and stress in five to eight-year-old children undergoing brief invasive dental treatments. Utilizing the VR gadget, the youngsters in the research were subjected to a VR distraction. The gadget is made up of an eyewear apparatus that projects the desired visuals in front of the child's eyes and occluding eye-pads that restrict his/her visual field from the dental surroundings. The device also had earbuds that minimized noise from the dental operatory and produced sounds from the virtual world. The findings indicate that VR distraction might be utilized as a beneficial behavioral change approach in children having minor dental procedures [18,19].

According to the research, VR is a form of technology that might help improve teenage studying of how to regulate emotions within human immunodeficiency virus prevention interventions as well as health promotion strategies that focus on feelings regulation skills, such as those that address food allergies, diabetes, as well as obesity [20].

## Conclusions

This case study intends to emphasize the need for early assessment and therapy and, most significantly, reducing sadness and stress as well as behavioral issues among children via the use of VR environmental video games. Immersive gaming using VR has been found to successfully alleviate sadness and anxiousness in heart condition sufferers. As a result, gaming in an immersive VR environment may be a potential strategy for reducing anxiety in people with congenital heart conditions.
